# GLP-1 agonist liraglutide decreases operant methamphetamine intake in rats under conditions of short- but not extended-access to the drug

**DOI:** 10.3389/fphar.2026.1791206

**Published:** 2026-05-01

**Authors:** Maria Hrickova, Sefa Furkan Demirci, Petra Amchova, Jana Ruda-Kucerova

**Affiliations:** Department of Pharmacology, Faculty of Medicine, Masaryk University, Brno, Czechia

**Keywords:** extended-access, GLP-1 agonist, intravenous self-administration, liraglutide, methamphetamine, rats

## Abstract

**Objective:**

Glucagon-like peptide-1 (GLP-1) receptor agonists have emerged as a therapeutic strategy for reducing drug craving and intake. However, their efficacy in methamphetamine use disorder remains unexplored. This study assessed the effects of repeated liraglutide treatment on methamphetamine intravenous self-administration in rats.

**Methods:**

Male Wistar rats were trained to self-administer methamphetamine in either short- (1.5-h) or extended- (6-h) access sessions. Once a stable level of drug intake was achieved, the animals received daily subcutaneous injections of liraglutide (0.1 mg/kg) or saline for 7 days, administered 1 hour before the session.

**Results:**

Operant responding and methamphetamine intake were significantly higher in the extended-access protocol, and the drug intake showed an escalation in time. A single administration of liraglutide did not affect methamphetamine intake in either the short- or extended-access paradigms. Following repeated administration, liraglutide significantly decreased methamphetamine intake in the short-but not in the extended-access study.

**Conclusion:**

The findings suggest that the GLP-1 receptor agonist liraglutide can reduce methamphetamine-taking behavior under conditions of low intake, whereas in the extended-access protocol, where drug intake escalates, the treatment shows no effect. Further research should better characterize its optimal dosing, duration of action, and brain penetration in rats.

## 1 Introduction

Glucagon-like peptide-1 (GLP-1) is a hormone produced in the gut and the brain, serving as a key communicator between these systems. It plays a crucial role in slowing gastric emptying, stimulating insulin production, and reducing food cravings and intake (Holst, 2007). Due to these effects, GLP-1 receptor agonists have been approved for the treatment of diabetes mellitus (Yao et al., 2024) and obesity (Popoviciu et al., 2023). In addition to their metabolic actions, GLP-1 receptor agonists modulate neural pathways involved in appetite regulation. More than a quarter of a century ago, research discovered that the precursor for GLP-1 is produced in the central nervous system, with GLP-1 receptors distributed across multiple brain regions in rats, including the mesolimbic system, ventral tegmental area (VTA) and nucleus accumbens (NAc) (Merchenthaler et al., 1999). This suggested a possible link between GLP-1’s appetite-suppressing effects and dopamine-based reward pathways. Early studies using immediate gene expression analysis indicated that GLP-1 and its analogs enhance neuronal activity in the mesolimbic system (Dossat et al., 2011). It was demonstrated that GLP-1 receptor agonists reduce progressive ratio operant responding for food and conditioned place preference (CPP) for chocolate pellets (Dickson et al., 2012).

Building on these findings, the investigation was quickly extended to drug-related rewards, and a growing body of preclinical studies across a range of substances suggests that GLP-1 receptor agonists can reduce behavioral patterns associated with addiction. A key hypothesis suggests this system reduces cravings by modulating dopamine-mediated reward processing. Mesolimbic reward areas receive direct innervation from GLP-1-producing neurons, indicating hindbrain-derived GLP-1 may influence reward-related behaviors (Richard et al., 2015; Trapp and Cork, 2015). GLP-1 receptor agonists modulate dopaminergic signaling by increasing dopamine-β-hydroxylase levels, reducing dopamine release, enhancing dopamine transporter expression, increasing dopamine turnover, and suppressing NAc projections, which attenuates phasic dopaminergic signaling and reduces consummatory behaviors (Eren-Yazicioglu et al., 2021). GLP-1 receptor activation reduces cocaine-induced dopamine levels in the NAc (Egecioglu et al., 2013b; Sørensen et al., 2015; Fortin and Roitman, 2017) with similar effects on cocaine, amphetamine (Egecioglu et al., 2013b), ethanol (Sørensen et al., 2016; Vallöf et al., 2016; 2019), and nicotine (Egecioglu et al., 2013a). Alcohol is the most extensively studied substance in this context, but GLP-1 agonists have also been investigated for their potential effects on nicotine, opioid, and stimulant use disorders (Bruns et al., 2024).

In terms of clinical evidence, randomized clinical trials have shown that the GLP-1 receptor agonist exenatide may reduce alcohol consumption, particularly in individuals with co-occurring alcohol use disorder and obesity (Klausen et al., 2022). For nicotine, studies examining GLP-1 receptor agonists exenatide and dulaglutide have demonstrated effects on post-cessation weight gain; however, findings related to smoking rates and long-term abstinence have been mixed (Yammine et al., 2021; Lengsfeld et al., 2023; Lüthi et al., 2024). Other GLP-1 receptor agonists, including liraglutide and semaglutide, are now being evaluated clinically across several substance use disorder domains (alcohol, nicotine, cocaine, and opioid use disorder), with multiple trials ongoing (NCT05895643, NCT07227948, NCT06924697 at ClinicalTrials.gov) or recently completed (Klausen et al., 2022; Freet et al., 2025). Yet, early clinical findings for opioid use disorder are promising, with a small randomized, double-blind, placebo-controlled residential study reporting an ∼40% reduction in opioid craving with liraglutide relative to placebo (Freet et al., 2025). However, robust randomized clinical trials are needed to fully understand the benefits of this treatment, and to date, there is no study focused on amphetamines.

Given the lack of evidence supporting a potential effect of GLP-1 receptor agonists as treatment in methamphetamine (METH) dependence in either a clinical or preclinical setting, the aim of this study was to investigate whether administration of liraglutide could reduce METH intake in a rat model of intravenous self-administration (IVSA). The study employed both short-access and extended-access paradigms, the latter of which is thought to more accurately reflect real-world drug exposure. Liraglutide was selected as the GLP-1 receptor agonist based on pharmacokinetic properties providing stable drug levels during extended-access sessions (Oh et al., 2023) and its widespread clinical use, enhancing the translational relevance of the present design. Moreover, a repeated liraglutide treatment regimen was used to better mimic clinical conditions, as significant therapeutic effects in chronic disorders such as addiction are rarely observed after a single exposure.

## 2 Materials and methods

### 2.1 Animals

**FIGURE 1:**
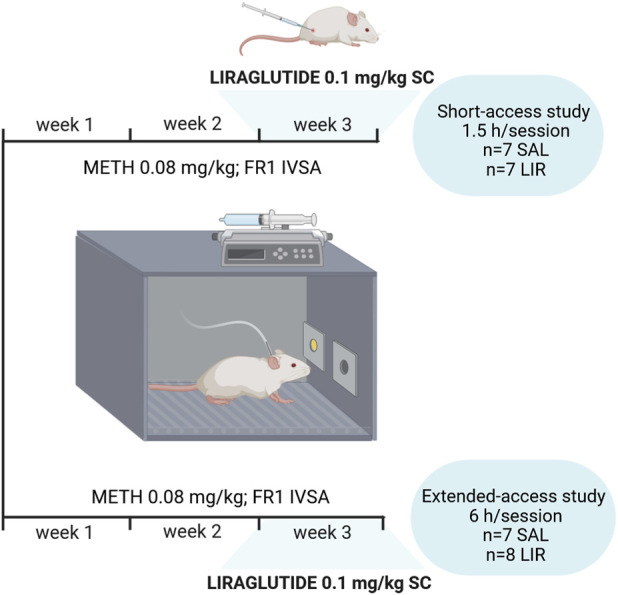
Timeline of the study. The figure indicates the design of both ShA and ExA self-administration experiments. There were 2 weeks of METH self-administration before initiation of the LIR treatment, which lasted 1 week.

A total of forty-eight adult male albino Wistar rats, within a weight range of 300–350 g start of the experiments, were obtained from the Masaryk University breeding facility (Brno, Czech Republic). The rats were initially pair-housed in standard open plastic cages. Following surgical procedures, the rats were housed individually with environmental enrichment provided in the form of a 15 cm long and 7.5 cm wide grey plastic tube. Some animals were lost during surgery, and the final cohort was selected based on the patency of their catheters. The final sample size consisted of 14 rats for the METH short-access study and 15 rats for the extended-access study. The experimental design is depicted in Figure 1.

Environmental conditions were maintained as follows: relative humidity of 50%-60%, room temperature of 22 ± 1 °C, and an inverted 12-h light-dark cycle (dark phase: 6 a.m. to 6 p.m.). Food and water were available *ad libitum* throughout the study. To track food consumption, standard rodent chow was administered at 50 g/day, and food intake was recorded daily by weighing the remaining amount and refilling the feeder daily.

All procedures were performed in accordance with the EU Directive No. 2010/63/EU and approved by the Animal Care Committee of the Faculty of Medicine, Masaryk University, Czech Republic, and the Czech Governmental Animal Care Committee, in compliance with the Czech Animal Protection Act No. 246/1992. Animal studies are reported in compliance with the ARRIVE guidelines (Lilley et al., 2020).

### 2.2 Drugs and treatments

Methamphetamine (METH) was purchased from Sigma Chemical Co. (St. Louis, MO, USA) and diluted in saline to achieve a concentration of 0.08 mg/kg per infusion, a dosage routinely used and validated in operant IVSA studies in our laboratory (Babinska et al., 2016; Ruda-Kucerova et al., 2017; 2021; Hrickova et al., 2024).

Liraglutide (LIR) was obtained from a local pharmacy as the human medication Victoza ® (6 mg/mL injectable solution in a prefilled pen), Novo Nordisk A/S, Denmark. The original solution was diluted with saline to achieve a final concentration of 0.1 mg/mL. LIR was administered subcutaneously 1 h before each session at a dose of 0.1 mg/kg. The treatment was administered for seven consecutive days. The timing and dosing of liraglutide were selected based on prior rodent self-administration studies, e.g., heroin and alcohol models (Marty et al., 2020; Douton et al., 2022a; 2022b), and on the known pharmacokinetic profile of liraglutide in rats, which shows peak plasma concentrations approximately 2–6 h after subcutaneous administration and brain penetration within 1 h (Oh et al., 2023).

### 2.3 Operant self-administration training

Operant training was initiated prior to catheter implantation using a food self-administration protocol to establish operant behavior in the animals. Ten operant conditioning boxes (30 × 25 × 30 cm, Coulbourn Instruments, USA) were utilized, each equipped with two nose-poke holes on one side and controlled by Graphic State Notation four software (Coulbourn Instruments, USA). Training was conducted under a fixed ratio 1 (FR-1) schedule of reinforcement, where a single nose-poke in the active hole resulted in the delivery of a palatable pellet (BioServ, sweet dustless rodent pellets, F0021-Purified Casein-Based Formula, 45 mg), containing 276 g/kg of monosaccharides and 310 g/kg of sucrose. Each session lasted 30 min and occurred during the dark phase of the inverted light-dark cycle for five consecutive days. During the sessions, a house light illuminated the cage, while a cue light in the active nose-poke hole indicated the availability of the pellets.

### 2.4 Implantation of the intrajugular catheters

Animals were anesthetized using isoflurane inhalation. A permanent intracardiac catheter was surgically implanted *via* the external jugular vein, reaching the right atrium. The external portion of the catheter was positioned to exit the skin in the midscapular region. Postoperative pain management was administered using meloxicam (2 mg/kg s.c.). A recovery period of 1 week was observed prior to the commencement of METH self-administration. To prevent infection and catheter occlusion, the catheters were flushed daily with an enrofloxacin solution (17 mg/kg), followed by 0.1 mL of a heparinized (1%) sterile saline solution. The procedure was previously established, validated, and repeatedly used (Babinska and Ruda-Kucerova, 2017; Ruda-Kucerova et al., 2017; Amchova and Ruda-Kucerova, 2023; Hrickova et al., 2024; 2025).

### 2.5 Operant self-administration of METH

Intravenous self-administration (IVSA) training was conducted within the same operant chambers as food training, utilizing a fixed-ratio 1 (FR-1) schedule. A nose-poke in the designated active hole triggered the infusion pump to administer a METH infusion, followed by a 20-s time-out period during which additional nose-pokes were recorded but not reinforced. Environmental cues included the illumination of the active nose-poke light to signal drug availability, while both the house light and cue light were extinguished during the time-out period. In the short-access (ShA) study, sessions lasted 1.5 h, while in the extended-access (ExA) study, sessions lasted 6 h. Sessions were conducted daily (7 days per week) between 9 a.m. and 3 p.m. during the dark phase of the inverted light-dark cycle. After 2 weeks of METH maintenance, a seven-day LIR treatment regimen was initiated, with control rats receiving saline injections.

### 2.6 Statistical data analysis

Data are presented as mean ± standard error of the mean (SEM). Normality was assessed using the Shapiro–Wilk test, and model residuals met assumptions for parametric analysis. Acute treatment effects were analyzed using unpaired Student’s t-tests. The effects of repeated treatment administration were analyzed using linear mixed-effects models for repeated measures. For the analysis of ShA vs. ExA data, the day of the session (14 days) and type of study (ShA or ExA) were included as fixed effects, along with their interaction (day*study). The subject was included as a random effect to account for within-subject correlations across repeated measurements. When significant interaction was detected, post hoc comparisons were performed using Fisher’s least significant difference (LSD) tests. Similarly, for the analysis of treatment effect within the ShA or ExA studies, an analogous method was employed: the day of the session (7 days) and type of treatment (LIR or SAL) were included as fixed effects, along with their interaction (day*treatment). All statistical analyses were performed using IBM SPSS Statistics (version 25). A two-tailed p-value <0.05 was considered statistically significant.

## 3 Results

### 3.1 Effect of liraglutide on body weight gain and food consumption

To evaluate the pharmacological effects of LIR, we examined daily body weight (BW) gain and food intake from days 8–21 of the self-administration period.

In the ShA study, the mixed model analysis identified significant effects of the LIR treatment (F1,168 = 121.21, p < 0.001), day of the study (F13,168 = 14.09, p < 0.001), and their interaction (F13,168 = 8.22, p < 0.001) on BW gain. The LSD post-hoc test for the interaction indicated that LIR was associated with reduced BW gain from the first day of treatment (day 15, p = 0.016) until the conclusion of the study (days 16-21, p < 0.001). A comparable effect was observed in the analysis of daily food intake, where treatment (F_1,168_ = 127.25, p < 0.001), day of the study (F_13,168_ = 7.79, p < 0.001), and their interaction (F_13,168_ = 10.98, p < 0.001) were significant. The LSD post-hoc test for the interaction revealed that LIR was associated with decreased food intake from the first day of treatment (day 15, p = 0.032) until the end of the study (days 16-18 and 20, p < 0.001; day 19, p = 0.005; day 21, p = 0.001). Additionally, a significant, albeit random, effect was observed prior to the initiation of treatment on day 12 (p = 0.037).

**FIGURE 2:**
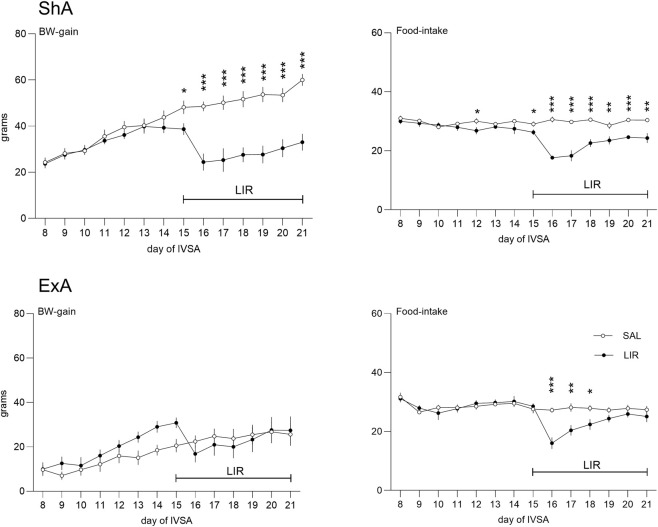
Effect of LIR on body weight gain and food intake. The line graphs show the mean ± SEM of body weight gain and food intake during days 8–21 (weeks 2-3) of METH IVSA, revealing the effect of LIR treatment. Data are shown for the short-access paradigm (1.5 h/session; upper graphs) and the extended-access paradigm (6 h/session; lower graphs); statistical comparison of SAL vs. LIR treated groups: *p < 0.05, **p < 0.01, ***p < 0.001.

In the ExA study, the mixed model analysis of the BW gain data revealed a significant effect solely for the day of treatment (F_13,150_ = 7.24, p < 0.001), which is not the primary focus of this investigation, and no significant effect for the treatment was observed. Conversely, the analysis of food intake demonstrated significant effects for the LIR treatment (F_1,150_ = 16.53, p < 0.001), day of the study (F_13,150_ = 6.21, p < 0.001), and their interaction (F_13,150_ = 3.39, p < 0.001). The LSD post-hoc test for the interaction indicated that LIR was associated with reduced food intake starting from the second day of treatment (day 16, p < 0.001), with this effect persisting for an additional 2 days (day 17, p = 0.001; day 18, p = 0.015) before returning to baseline levels. All data are illustrated in Figure 2.

### 3.2 ShA vs. ExA operant behavior

**FIGURE 3:**
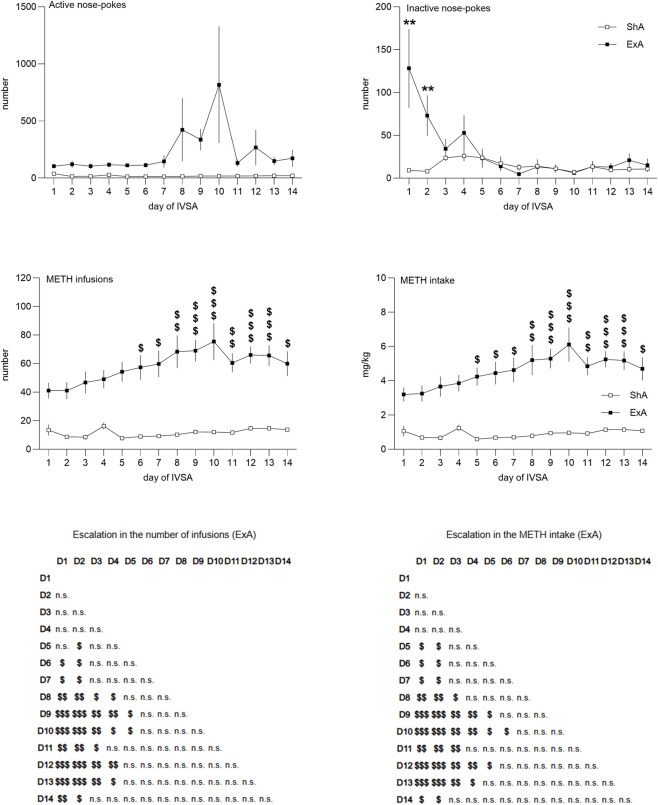
Operant training behavior before LIR treatment. The line graphs illustrate a comparative analysis of the short-access paradigm (1.5 h/session) and the extended-access paradigm (6 h/session), depicted as the mean ± SEM of active and inactive nose-pokes, the number of METH infusions, and METH intake (mg/kg per session). Statistical comparisons between the ShA and ExA groups revealed significantly higher scores across all variables in the ExA group (main effect not depicted in the graphs). Post-hoc analysis indicated increased inactive responding in the ExA group during the initial 2 days (**p < 0.01). Further post-hoc analysis for the number of infusions and METH intake demonstrated an escalation in drug consumption in the ExA group. The $ symbols in the graphs denote comparisons exclusively between day 1 and subsequent days of operant training in the ExA animals ($p < 0.05, $$p < 0.01, $$$p < 0.001). The tables beneath the METH infusion and METH intake graphs provide comprehensive statistical results within the respective datasets, encompassing all possible comparisons. Pairwise statistical comparisons of METH intake and number of infusions across experimental days (D1–D14) are presented in a matrix format. Each cell represents the comparison between the corresponding 2 days ($p < 0.05, $$p < 0.01, $$$p < 0.001, n. s. – not significant).

Figure 3 illustrates the primary characteristics of operant behavior in the ShA and ExA studies. The parameters assessed during the maintenance of METH intravenous self-administration (IVSA) included the number of active and inactive nose-pokes, the total number of METH injections received, and the dose of self-administered METH (in mg/kg). All variables were significantly higher in the ExA study, specifically active nose pokes showed only the main effect (F_1,26_ = 23.82, p < 0.001), while inactive nose pokes showed not only the main effect (F_1,26_ = 15.63, p < 0.001), but also the study*day interaction (F_1,26_ = 2.70, p < 0.001) and the LSD post hoc indicated a significantly higher inactive nose poking on the first 2 days of the ExA study compared to the ShA study (day 1: p = 0.004, day 2: p = 0.002). No difference between the studies in the remaining 12 days indicates successful learning of the operant behavior. The analysis of the number of infusions showed the main effect (F_1,26_ = 505.51, p < 0.001) and the study*day interaction (F_1,26_ = 2.18, p < 0.001). The post hoc testing for the study*day interaction revealed an escalation of the received number of infusions in the ExA but not the ShA study. Analogous findings were observed in the METH intake data: main effect (F_1,26_ = 526.31, p < 0.001) and the study*day interaction (F_1,26_ = 2.29, p < 0.001). The average (mean ± SD) METH consumption during the second week of operant training was 1.00 ± 0.38 mg/kg in the ShA study and 5.23 ± 2.30 mg/kg in the ExA study. The post-hoc testing for the study*day interaction revealed many significant differences in both variables–number of infusions and drug intake–as depicted in Figure 3.

### 3.3 Effect of liraglutide on METH intake

The acute LIR effect was evaluated on day 15 of the operant training. In either study, no significant effect on total intake was detected. In the ShA study, control rats self-administered 1.26 ± 0.11 mg/kg METH, whereas LIR-treated rats self-administered 0.95 ± 0.10 mg/kg METH (mean ± SEM). The t-test did not show a significant difference (p = 0.075). In the ExA study, the METH intake after an acute treatment was 5.41 ± 1.05 mg/kg in control and 4.72 ± 0.71 mg/kg in LIR-treated rats (t-test, p = 0.625).

The analysis of repeated treatment in the ShA study showed a significant effect of LIR treatment on active responding (F1,81 = 8.19, p = 0.005), indicating fewer active nose-pokes in LIR-treated rats. Conversely, LIR treatment increased inactive responding (F1,81 = 16.59, p < 0.001). This effect rules out the non-specific effect of the treatment on operant behavior. Furthermore, LIR was found to decrease daily METH intake (F1,81 = 29.97, p < 0.001). In any of these variables, the main effect did not interact with the day of the study. In the analysis of cumulative METH intake, significant effects of the LIR treatment (F_1,77_ = 47.21, p < 0.001), day of the study (F_6,77_ = 96.40, p < 0.001), and their interaction (F_6,77_ = 3.97, p = 0.002) were observed. The LSD post-hoc test for the interaction revealed that LIR was associated with cumulative METH intake from the second day of the treatment (day 16, p = 0.011) till the end of the study (day 17, p = 0.005; day 18, p = 0.007; days 19-21, p = 0.004).

The analysis of repeated treatment in the ExA study showed no significant effect of LIR treatment on active responding (F1,70 = 0.46, p = 0.499), inactive responding (F1,70 = 3.60, p = 0.062), daily (F1,70 = 2.11, p = 0.151), or cumulative METH intake (F1,70 = 2.33, p = 0.131). In any of these variables, the main effect did not interact with the day of the study.

**FIGURE 4:**
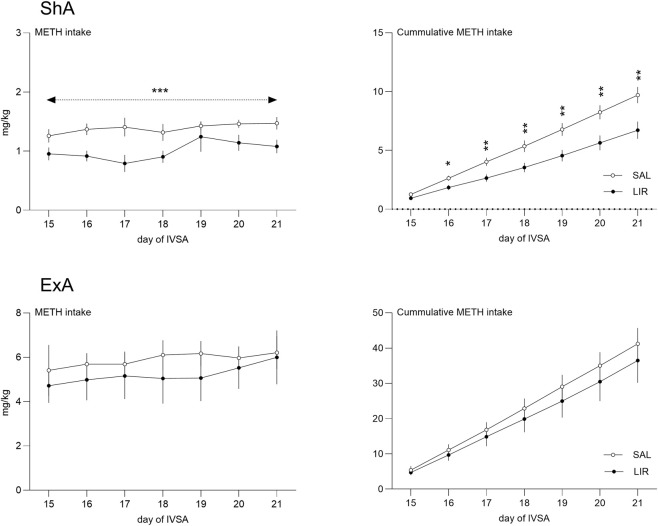
Effect of repeated LIR treatment on METH intake. Effects of repeated LIR treatment on METH intake and cumulative METH intake across 7 days of treatment during METH IVSA. Data are presented for the short-access paradigm (1.5 h/session; upper panels) and the extended-access paradigm (6 h/session; lower panels); *p < 0.05, **p < 0.01, ***p < 0.001.

The data on METH intake are depicted in Figure 4.

## 4 Discussion

Repeated administration of LIR significantly reduced body weight gain and food consumption in rats subjected to the short-access METH self-administration paradigm. The anorectic and weight-suppressing effects were evident from the first day of liraglutide treatment and persisted throughout the seven-day dosing period. Conversely, in the extended-access paradigm, LIR only temporarily reduced food intake during the first three treatment days, without significantly affecting body weight gain. These findings align with LIR’s known metabolic profile and confirm that the administered dose exerted pharmacological effects on appetite regulation and energy balance, thereby confirming treatment efficacy during behavioral testing. Similar findings were previously reported (Douton et al., 2022b). The absence of the LIR effect in the extended-access study can be attributed to the anorectic effects of high doses of METH itself (Bittner et al., 1981). A marked difference in body weight gain is already apparent by day 8 (after the first week of METH operant intake): the short-access group gained over 20 grams, while the extended-access group increased by 10 grams, despite identical food intake. The additive effect of LIR over METH on the body weight gain in the extended-access cohort is also evident in the graph, although it did not reach statistical significance. This may be due to a non-significant yet apparent basal difference in the BW gain in the LIR and SAL-treated groups. This basal difference occurred by chance; the rats were already assigned to the treatment groups at the beginning of the experiment.

Regarding METH intake, repeated LIR administration led to a significant and sustained reduction within the short-access model, but not under extended-access conditions. After repeated administration, rats in the short-access paradigm exhibited lower daily and cumulative METH intake than saline controls, with this effect becoming apparent on the second day of treatment and continuing throughout the study, while LIR did not affect METH-taking behavior in the extended-access paradigm. Notably, this effect cannot be attributed to a non-specific suppression of operant behavior as visible in higher inactive responding scores in the short-access and unaltered behavior in the extended-access model.

In preclinical studies, systemic administration of GLP-1 receptor agonists has been shown to reduce cocaine-induced CPP (Egecioglu et al., 2013b; Graham et al., 2013) and its operant self-administration (Sørensen et al., 2015), while less research has focused on their effects on amphetamine intake. Available data suggest that they can reduce amphetamine-induced hyperlocomotion and CPP (Erreger et al., 2012; Egecioglu et al., 2013b) and similar effects have been reported for DPP-4 inhibitors (Lautar et al., 2005). These findings suggest a potential role of GLP-1 signaling in addiction-related behaviors, likely mediated by central mechanisms. *In vivo* microdialysis studies have shown that GLP-1 receptor activation reduces dopamine release in reward-related regions such as NAc, indicating a suppression of dopaminergic reinforcement mechanisms (Egecioglu et al., 2013b; Sørensen et al., 2015). Furthermore, intra-VTA and intra-NAc administration of GLP1 attenuates cocaine self-administration (Schmidt et al., 2016) and seeking (Hernandez et al., 2018; 2019). These findings together highlight a central role for GLP-1 signaling in modulating the reinforcing and relapse-related effects of psychostimulants.

The doses used in some of these studies also produced non-specific behavioral effects, such as reduced locomotor activity or induced malaise (Egecioglu et al., 2013b; Sørensen et al., 2015), which may bias the behavioral outcomes (Kanoski et al., 2012), which may have contributed to the reported reductions in drug-related behaviors (Egecioglu et al., 2013b; Graham et al., 2013; Sørensen et al., 2015). However, the reduction in the METH intake observed in the present study cannot be solely attributed to a general suppression of operant behavior, because inactive nose-poke responding increased following liraglutide treatment in the short-access, while in the extended-access study, this effect was not evident. Moreover, a generalized reduction in motivation or malaise would be expected to increase latency to the first infusion, which was not observed in either study. While our data do not support a general reduction in motivation, a clear dissociation between drug-directed and general motivational effects would require additional control conditions such as non-drug saline IVSA.

Regarding the lack of liraglutide effect in the extended-access study, this model precipitates neuroplastic changes in dopamine and glutamate signaling within reward circuits, which may reduce the sensitivity to GLP-1 receptor agonist intervention (Rogers et al., 2008; Yuan et al., 2011; Poisson et al., 2021). The escalation of drug intake and reinforced drug-seeking behaviors in extended-access models may represent a stage of METH-induced neurobiological adaptations that are less modulated by GLP-1 signaling pathways. In contrast to the short-access model, rats in the extended-access protocol may exhibit reduced sensitivity to LIR’s modulation of dopamine-driven reinforcement, suggesting that more intensive or selective interventions would be needed to produce a behavioral effect. Besides, an altered metabolic state under extended METH access could also play a role. METH is known to alter metabolism extensively in the brain and periphery (Kim et al., 2019), potentially altering the pharmacodynamic and pharmacokinetic properties of any xenobiotic.

Another key issue in this line of research is the pharmacokinetic properties of LIR in rats, including absolute bioavailability and brain penetration, as well as typical side effects of the GLP-1 receptor agonist class, such as nausea. Species-specific pharmacokinetic profiles of LIR could impact its bioavailability in the brain and its T-max (time to reach maximum plasma concentration after administration), which may influence dosage intervals and its overall effect. In preclinical research on drug addiction and the GLP-1 system, most studies have used exendin-4, a short-acting GLP-1 receptor agonist, as a treatment. Some studies have also explored the effects of longer-acting analogs, such as LIR (Brunchmann et al., 2019; Eren-Yazicioglu et al., 2021; Bruns et al., 2024). We also chose LIR, a longer-acting GLP-1 agonist, to reduce fluctuations in plasma levels and observe its effects over an extended study period (6 h), which should more closely mimic the human condition of drug exposure. However, the pharmacokinetics of LIR in rodents differ from those observed in humans. Notably, its half-life has been reported to be around 4 h in rats (Sturis et al., 2003), suggesting that it may not act as long in animals as it does in humans. However, pharmacokinetic data indicate that a 0.03 mg dose of LIR reaches its maximum concentration (T-max) approximately 8 h after administration, with behavioral effects detectable by 6 h (Douton et al., 2022a). LIR has also been detected in the hypothalamus within 4 h following intravenous injection (Secher et al., 2014). Hence, it seems that pharmacokinetic constraints are not responsible for the different outcomes of the short- and extended-access studies. Nevertheless, comprehensive pharmacokinetic studies in rodents are still lacking, making it difficult to draw firm conclusions.

Another aspect to consider is the ability of GLP-1 ligands to cross the blood-brain barrier (BBB). As peptide-based molecules, GLP-1 agonists are relatively large and do not readily diffuse across the BBB. In rodent studies, exendin-4 and dulaglutide have been detected in the brain following systemic administration, supporting their ability to engage central GLP-1 receptors, and these compounds are generally considered to be capable of penetrating the BBB (Ørskov et al., 1996; Kastin et al., 2002; Hernandez et al., 2018; 2019; Dong et al., 2022; Oh et al., 2023; Rhea et al., 2024). Although existing literature suggests that larger GLP-1 receptor agonists such as LIR and semaglutide do not enter the brain, some studies have challenged this view (Bruns VI et al., 2024; Christensen et al., 2015). For example, systemically injected LIR was detected in the hypothalamus, cerebellum, and brain stem (Secher et al., 2014; Salinas et al., 2018; Oh et al., 2023). LIR was also shown to cross the BBB following intraperitoneal administration at 25 and 250 nmol/kg, while no measurable increase in brain levels was detectable at the lowest tested dose, 30 min post-injection, nor at a later time point (3 h), even with the highest dose suggesting that LIR’s brain penetration is both dose- and time-dependent (Hunter and Hölscher, 2012). Another important consideration in this regard is that drugs of abuse may compromise the integrity of the BBB, potentially increasing its permeability. METH has been shown to acutely disrupt the BBB through direct effects on vascular endothelium and to chronically impair its function *via* neuroinflammatory and oxidative stress pathways (Ramirez et al., 2009; Turowski and Kenny, 2015; Sajja et al., 2016). Nevertheless, we observed an effect of LIR administered subcutaneously in a short-access paradigm, suggesting a potential therapeutic benefit under specific conditions.

Furthermore, nausea is among the most common side effects associated with GLP-1 receptor agonists in humans (Bettge et al., 2017). There is an ongoing debate concerning the extent to which the effects of GLP-1R agonists on alcohol and drug intake are mediated by visceral malaise and/or aversion rather than specific motivational mechanisms. This issue is similarly observed in animal models; specifically, exendin has been demonstrated to induce nausea-like effects in rodents in a dose-dependent manner, as assessed by conditioned taste aversion tests or pica responses. LIR has also been reported to induce malaise in rodents, although a dose of 0.05 mg/kg elicited effects only on the first day of treatment, as indicated by pica behavioral response (Kanoski et al., 2012). Moreover, similar findings have been reported in human clinical studies, where nausea is more pronounced during the early stages of treatment (Bettge et al., 2017). In the present study, we used a LIR dose of 0.1 mg/kg, which has been reported not to increase pica behavior in rats (Douton et al., 2022b). If this dose were to induce malaise, we would anticipate observing an acute effect of the treatment, which was not present. Therefore, it is unlikely that this dose induced sufficient malaise to reduce drug craving. Importantly, this dose has also been reported not to impair motor activity (Douton et al., 2022a), or alter insulin or plasma glucose levels (Evans et al., 2022). ​

A limitation of the present study is the use of male rats, as sex-dependent differences have been reported across different stages of METH addiction in both humans and rodents (Fattore et al., 2008). Moreover, sex-dependent differences in the effects of GLP-1 receptor agonists have been reported clinically, with some evidence suggesting greater weight-loss responses and potentially different pharmacokinetic profiles in women and men (Yang et al., 2025), whereas preclinical studies have shown stronger suppression of alcohol self-administration and reinstatement in males, with females exhibiting less pronounced behavioral effects (Díaz-Megido and Thomsen, 2023). Another study using the long-acting GLP-1 receptor agonist dulaglutide reported sex-dependent effects on alcohol consumption, with stronger and more persistent reductions in males and different alterations in reward-related brain signaling between males and females (Vallöf et al., 2020). Taken together, existing sex differences in METH-induced behaviors, as well as the extent of the pharmacological effects of GLP-1 receptor agonists, may lead to different outcomes in female subjects.

In conclusion, this study demonstrates that repeated administration of the GLP-1 receptor agonist liraglutide significantly reduces METH intake during the maintenance phase of operant intravenous self-administration in a short-access model. However, this effect was not observed in the extended-access paradigm. The findings show certain benefits of GLP-1 receptor agonists for treating METH use disorder. However, the differential effects observed between short-access and extended-access models highlight the complexity of drug-seeking behaviors and warrant further research on the potential effects of METH-induced metabolic changes across the whole body (Kim et al., 2019). This experimental study did not investigate a complete dose-response effect of LIR; rather, it employed a single dose. This decision was informed by the documented adverse gastrointestinal effects associated with higher doses of this GLP-1 agonist, as indicated by pica behavior (Kanoski et al., 2012) and the known effect of this dosing in heroin and alcohol models (Marty et al., 2020; Douton et al., 2022a; 2022b).

Future studies should aim to replicate the findings, ideally with a larger sample size to increase statistical power; explore other GLP-1 receptor agonists, given their diverse pharmacokinetic and pharmacodynamic properties; and incorporate a wider range of operant designs (e.g., progressive-ratio schedules, reinstatement paradigms). In addition, to clarify whether the observed reduction in METH intake reflects a specific effect on rewarding operant behavior or a more general reduction in motivation or activity, additional behavioral measures such as conditioned aversion and locomotor activity should be employed. Furthermore, there is a lack of data in female subjects, using different METH and GLP-1 receptor agonist dosing, and more types of operant designs with more subject and (progressive protocols, reinstatement studies, etc.). Additionally, investigating the optimal dosing, duration of action, and brain penetration of these compounds will be crucial in developing effective treatments for METH addiction. The clinical relevance of these findings is enhanced by the existing approval of GLP-1 receptor agonists for other indications. The development of oral formulations and novel small GLP-1 agonists may further improve treatment adherence and efficacy.

## Data Availability

The raw data supporting the conclusions of this article will be made available by the authors, without undue reservation.
